# Elevated hemoglobin glycation index identify non-diabetic individuals at increased risk of kidney dysfunction

**DOI:** 10.18632/oncotarget.18572

**Published:** 2017-06-19

**Authors:** Teresa Vanessa Fiorentino, Maria Adelaide Marini, Elena Succurro, Angela Sciacqua, Francesco Andreozzi, Francesco Perticone, Giorgio Sesti

**Affiliations:** ^1^ Department of Medical and Surgical Sciences, Viale Europa, University Magna Græcia of Catanzaro, 88100 Catanzaro, Italy; ^2^ Department of Systems Medicine, University of Rome Tor Vergata, 00133 Rome, Italy

**Keywords:** non-enzymatic protein glycation, kidney dysfunction, hemoglobin glycation index, chronic kidney disease

## Abstract

Hemoglobin glycation index (HGI), calculated as the difference between the observed value of HbA1 and the predicted HbA1c based on plasma glucose concentration, is a measure of the individual tendency toward non-enzymatic hemoglobin glycation which has been found to be positively associated with nephropathy in subjects with diabetes. In this cross-sectional study we aimed to evaluate whether higher HGI levels are associated with impaired kidney function also among nondiabetic individuals.

The study group comprised 1505 White nondiabetic individuals stratified in quartiles according to HGI levels. Estimated glomerular filtration rate (eGFR) was calculated by using the MDRD equation.

Individuals in the intermediate and high HGI groups exhibited a worse metabolic phenotype with increased levels of visceral obesity, total cholesterol, triglycerides, inflammatory biomarkers such as hsCRP and white blood cells count and lower values of HDL and insulin sensitivity assessed by Matsuda index in comparison to the lowest quartile of HGI. Subjects in the intermediate and high HGI groups displayed a graded decrease of eGFR levels in comparison with the lowest quartile of HGI. In a logistic regression analysis individuals in the highest quartile of HGI exhibited a significantly 3.6-fold increased risk of having chronic kidney disease (95% CI: 1.13–11.24, *P* = 0.03) and a significantly 1.6-fold increased risk of having a mildly reduced kidney function (95% CI: 1.19–2.28, *P* = 0.003) in comparison to individuals in the lowest HGI group.

In conclusion HGI may be a useful tool to identify nondiabetic individuals with an increased risk of having kidney dysfunction.

## INTRODUCTION

Renal dysfunction is becoming a major public health problem worldwide. The human and economic burden caused by this affection is growing as a consequence of its progression to the end stage renal disease, a condition requiring dialysis or kidney transplantation, and the associated cardiovascular morbidity and mortality [1–3]. Given these clinical implications, early identification of individuals with impaired renal function is highly warranted to counteract the progression of the disease and prevent clinical adverse outcomes [3].

Impaired glucose metabolism has been found to be related with kidney dysfunction [4–5] and several studies have demonstrated a correlation between glycated hemoglobin (HbA1c) and kidney disease in subjects affected by diabetes as well as in non-diabetic population [6–8]. HbA1c is a commonly used indicator of glycemic control in patients affected by diabetes and a well-established predictor of diabetic complications [9–11]. Additionally, measurement of HbA1c has been suggested by the International Expert Committee as a diagnostic criterion for diagnosis of diabetes and prediabetes conditions [11–13].

It is widely recognized that a strong relationship exists between HbA1c and blood glucose levels; however, a considerable inter-individual variability of HbA1c with regard to blood glucose levels has been observed in diabetic subjects as well as in nondiabetic individuals [14–22]. Individuals with HbA1c levels persistently higher or lower than those expected in consideration of their mean blood glucose levels have been identified and referred to as having a high or low hemoglobin glycation phenotype, respectively [14]. In an attempt to quantify the disparity between HbA1c and the other measures of blood glucose homeostasis, Hempe et al. developed a mathematical method which was termed hemoglobin glycation index (HGI) [14]. The HGI is calculated as the difference between the observed value of HbA1c and the one predicted on the basis of blood glucose levels, estimated by inserting plasma glucose concentration into a population regression equation expressing the linear association between HbA1c and plasma glucose levels [14, 17, 18]. HGI is a measure of the degree of hemoglobin glycation, and an independent association between HGI and microvascular diabetic complications has been reported in some studies [14, 17, 18]. In this regard, a higher value of HGI has been shown to be associated not only with prevalent nephropathy, but also with an increased risk to develop kidney disease in subjects with diabetes [17, 18].

However, whether higher HGI levels may identify subjects with a greater risk to have impaired kidney function also among nondiabetic individuals has not been investigated yet. The aim of this study was therefore to evaluate the link between the degree of hemoglobin glycation, estimated by HGI, and renal function in a large cohort of White subjects without diabetes.

## RESULTS

The whole study cohort comprised 1505 individuals, of whom 683 (45.5%) were male. The mean age was 47 ± 15 years and mean body mass index (BMI) was 29 ± 6 kg/m^2^. Table 1 shows the anthropometric and biochemical features of the study subjects stratified according to quartiles of HGI levels.

**Table 1:** Anthropometric and metabolic characteristics of the study subjects stratified according to hemoglobin glycation index

Variables	Whole study subjects	1 Quartile (–2.55;–0.16)	2 Quartile (–0.16;0.4)	3 Quartile (0.4;0.25)	4 Quartile (0.25;1.09)	*P*
Gender (*Male/Female*)	683/822	168/209	169/206	166/210	180/197	0.75
Age *(yrs)*	47 ± 15	43 ± 15	44 ± 14	48 ± 15 £££ ###	51 ± 13 £££ ### $$$	< 0.0001§
BMI *(kg/m2)*	29 ± 6	28.1 ± 5.7	28.9 ± 5.9	29.2 ± 5.6 £££	30.9 ± 6.6 £££ ### $$$	< 0.0001*
Waist circumference (*cm*)	99 ± 14	96 ± 13	98 ± 14	99 ± 15 ££	104 ± 16 £££ ### $$$	< 0.0001*
Fat Mass (*%*)	32.1 ± 10	31.1 ± 11	31.5 ± 10	32.1 ± 9	34.2 ± 8 £££ ## $	0.03*
Current smokers N. (*%*)	281 (22%)	57 (19%)	74 (22%)	63 (20%)	87 (27%) £	0.02
SBP *(mmHg)*	126 ± 17	125 ± 17	125 ± 17	125 ± 18	128 ± 16	0.13
DBP *(mmHg)*	78 ± 11	77 ± 11	78 ± 10	78 ± 11	79 ± 10	0.96
Fasting glucose (*mg/dl*)	92 ± 11	92 ± 11	92 ± 11	93 ± 10	92 ± 12	0.16
1-h post-load glucose (*mg/dl*)	150 ± 43	145 ± 45	146 ± 40	150 ± 43	161 ± 43	0.21
2-h post-load glucose (*mg/dl*)	120 ± 31	114 ± 30	118 ± 31	121 ± 31	128 ± 32	0.11
Fasting insulin *(µU/ml)*	13 ± 9	12 ± 7	13 ± 10	13 ± 8	15 ± 11	0.40
1-h insulin *(µU/ml)*	109 ± 85	96 ± 67	110 ± 96	110 ± 85	119 ± 92	0.77
2-h insulin *(µU/ml)*	96 ± 90	80 ± 73	96 ± 80	96 ± 84	116 ± 95 £££	0.08
Matsuda insulin sensitivity index (*mg x L*^2^ *x mmol*^-1^ *x mU*^-1^ *x min*^-1^)	74 ± 48	84 ± 49	78 ± 52	72 ± 47 ££	66 ± 45 £££	0.05
HbA1c (*%*)	5.4 ± 0.4	5.0 ± 0.3	5.4 ± 0.2 £££	5.6 ± 0.2 £££ ###	5.8 ± 0.2 £££ ### $$$	< 0.0001
Total cholesterol (*mg/dl*)	197 ± 38	191 ± 39	197 ± 37	200 ± 37 ££	201 ± 39 ££	0.03
HDL (*mg/dl*)	52 ± 14	53 ± 14	52 ± 14	51 ± 14 £	50 ± 15 ££ #	0.01
Triglycerides (*mg/dl*)	119 ± 67	108 ± 64	115 ± 65	121 ± 68 ££	130 ± 70 £££ #	0.01
hsCRP (*mg/l*)	3.0 ± 3	2.5 ± 3.0	2.9 ± 3.0	3.0 ± 3.0 £	3.7 ± 3.0 ££	0.03
WBC count (*x10*^9^*/l*)	6840 ± 1919	6163 ± 1517	6751 ± 1725 £££	6917 ± 2087 £££	7151 ± 1852 £££ ###	< 0.0001
Hematocrit (%)	42.3 ± 4.8	42.2 ± 4.2	42.6 ± 5.8	42.2 ± 4.3	42.9 ± 5.4	0.71
Hemoglobin (*g/l*)	13.9 ± 1.5	13.9 ± 1.5	13.9 ± 1.5	13.8 ± 1.5	13.9 ± 1.5	0.12
Metabolic syndrome, No (%)	425 (28%)	84 (22%)	91 (24%)	111 (29%) £	139 (36%) £££ # $	< 0.0001
ACE inhibitor or Angiotensin receptor blocker therapy, No (%)	440 (29%)	82 (21%)	89 (23%)	111 (29%) £	158 (41%) £££ ## $$	< 0.0001
Diuretics, No (%)	191 (12%)	27 (7%)	34 (9%)	51 (13%)	79 (20%) £££ ## $	< 0.0001
Calcium channel blockers, No (%)	147 (10%)	35 (9%)	35 (9%)	36 (10%)	41 (11%)	0.97
Statin therapy, No	146 (10%)	26 (7%)	29 (7%)	34 (9%)	57 (15%) £££ ### $$	0.001

Data are means ± SD. Fasting, 1-h, 2-h insulin, triglycerides, HDL, hsCRP, and ERS were log transformed for statistical analysis, but values in the table represent back transformation to the original scale. Categorical variables were compared by χ^2^ test. Comparisons between the four groups were performed using a general linear model for multiple comparisons. *P* values refer to results after analyses with adjustment for age, gender, and BMI. § *P* values refer to results after analyses with adjustment for gender. *P values refer to results after analyses with adjustment for gender and age.

BMI: body mass index; SBP: systolic blood pressure; DBP: diastolic blood pressure; hsCRP: high sensitivity C reactive protein; HDL: high density lipoprotein; ERS: erythrocytes sedimentation rate; WBC: white blood cell count; ACE: angiotensin-converting-enzyme.

£ *P* < 0.05 vs Quartile 1 of HGI; ££ *P* < 0.01 vs Quartile 1 of HGI; £££ *P* < 0.001 vs Quartile 1 of HGI.

# *P* < 0.05 vs Quartile 2 of HGI; ## *P* < 0.01 vs Quartile 2 of HGI; ### *P* < 0.001 vs Quartile 2 of HGI.

$ *P* < 0.05 vs Quartile 3 of HGI; $$ *P* < 0.01 vs Quartile 3 of HGI; $$$ *P* < 0.001 vs Quartile 3 of HGI

We found that age was significantly different between the four study groups; subjects in the high (quartile 4) and intermediate (quartile 3 and 2) HGI groups were older than individuals in the low HGI group (quartile 1). After adjusting for age and gender, significant difference between the four study groups were found with respect to BMI, waist circumference and fat mass, with a progressive increase of these adiposity measures in the intermediate and high HGI groups in comparison to low HGI subjects.

By design individuals in the intermediate and high HGI groups exhibited progressively higher levels of HbA1c in comparison to the lowest quartile of HGI, however no differences in fasting, 1 hour and 2 hour post-challenge glucose levels were detected between the four study groups.

After adjusting for age, gender and BMI, we found that higher HGI levels were associated with a worse cardio-metabolic phenotype. A higher proportion of subjects in the high HGI group were current smokers in comparison to the low HGI group. A graded increase in the levels of total cholesterol, triglycerides, and inflammatory biomarkers such as high sensitivity C reactive protein (hsCRP) and white blood cell (WBC) count was observed in the intermediate and high HGI groups in comparison with the lowest quartile of HGI. Furthermore, in comparison to individuals in the lowest HGI group, those in the intermediate and high HGI groups displayed a significant decrease in high density lipoprotein (HDL) cholesterol levels, and insulin sensitivity assessed by the Matsuda index. Subjects in the intermediate and high HGI groups were more likely to have metabolic syndrome. A higher proportion of subjects in the intermediate and high HGI groups was treated with angiotensin-converting-enzyme inhibitor (ACE) inhibitors and angiotensin receptor blockers. A higher proportion of subjects in the high HGI quartile was taking diuretics and statins. No significant difference between the study groups were detected in terms of systolic and diastolic blood pressure, hematocrit and hemoglobin levels.

**Figure 1:**
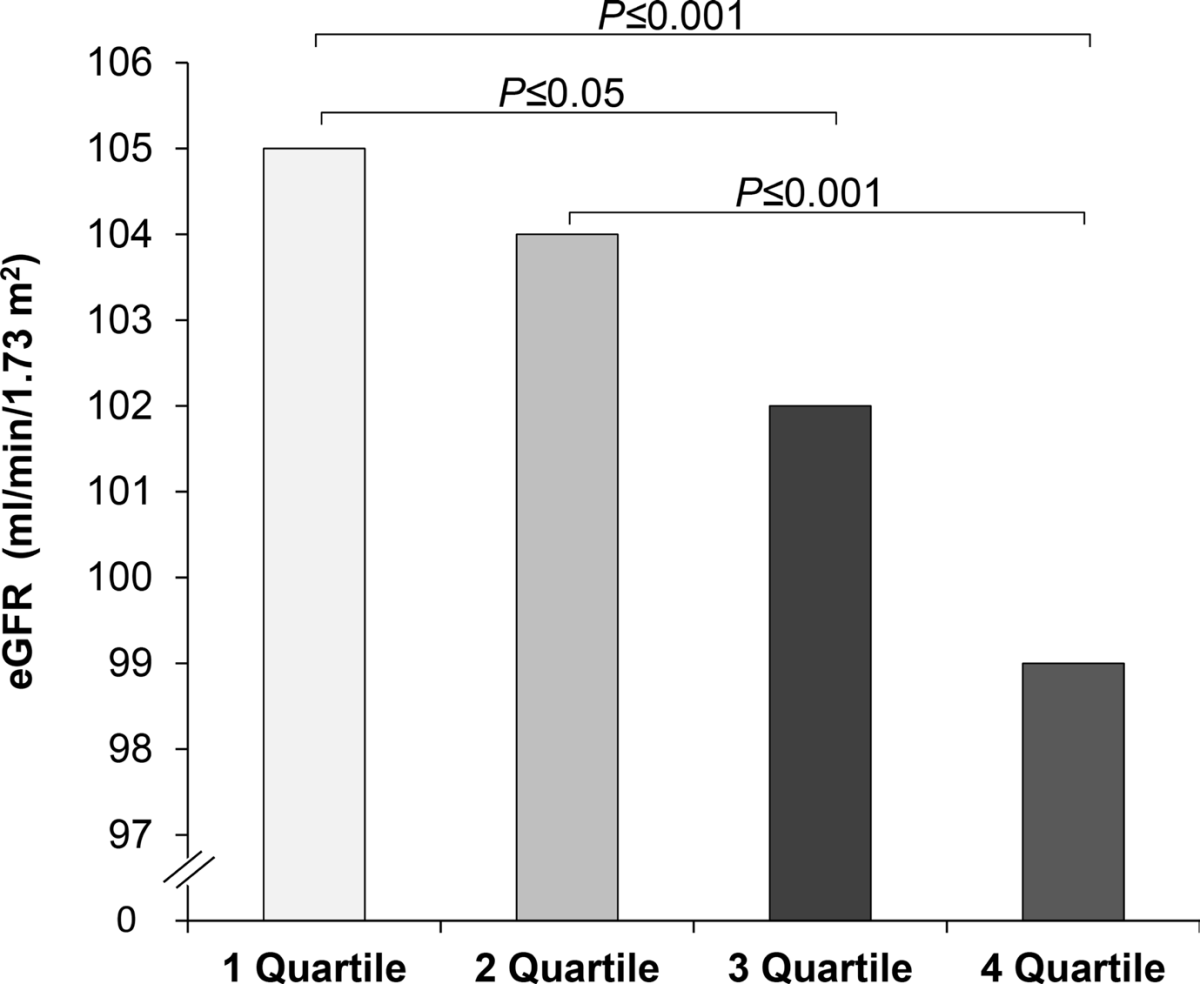
Estimated glomerular filtration rate (eGFR) in study participants stratified according to hemoglobin glycation index (HGI)

Notably, a significant and negative correlation between HGI and estimated glomerular filtration rate (eGFR) was observed, with subjects in the intermediate and high HGI groups exhibiting a graded decrease of eGFR levels in comparison with the lowest quartile of HGI (Table 2 and Figure 1). Moreover even after adjustment for metabolic syndrome diagnosis, anti-hypertensive treatments and statin therapy in addition to gender and BMI, subjects in the high HGI group displayed significantly lower levels of eGFR in comparison to the low HGI group (*P* ≤ 0.02).

**Table 2:** Renal function of the study subjects stratified according to hemoglobin glycation index

Variables	Whole study subjects	1 Quartile (–2.55;–0.16)	2 Quartile (–0.16;0.4)	3 Quartile (0.4;0.25)	4 Quartile (0.25;1.09)	*P*
Creatinine (*mg/dl*)	0.78 ± 0.17	0.76 ± 0.17	0.77 ± 0.17	0.78 ± 0.17	0.80 ± 0.22 ££	0.05
eGFR (*ml/min/1.73m*^2^)	107 ± 22	105 ± 23	104 ± 21	102 ± 22 £	99 ± 24 £££ ###	0.001
ACR (μg*/mg*) (*n* = 401)	14 ± 11	11 ± 9	13 ± 11	13 ± 11	18 ± 13 ££ # $	0.04
CKD (eGFR < 60 ml/min/1.73 m^2^)	27 (1.8%)	3 (0.8%)	4 (1%)	8 (2.1%) £	12 (3.2%) ££ #	0.02
Mildly reduced kidney function (eGFR: 90–60 ml/min/1.73 m^2^)	391 (26.1%)	88 (23%)	87 (23%)	93 (25%)	123 (33%) ££ ## $	0.001

Data are means ± SD. Categorical variables were compared by χ^2^ test. Comparisons between the four groups were performed using a general linear model for multiple comparisons. *P* values refer to results after analyses with adjustment for gender and BMI.

eGFR = estimated glomerular filtration rate; CKD = chronic kidney disease; ACR = spot urine albumin creatinine ratio.

£ *P* < 0.05 vs Quartile 1 of HGI; ££ *P* < 0.01 vs Quartile 1 of HGI; £££ *P* < 0.001 vs Quartile 1 of HGI.

# *P* < 0.05 vs Quartile 2 of HGI; ## *P* < 0.01 vs Quartile 2 of HGI; ### *P* < 0.001 vs Quartile 2 of HGI.

$ *P* < 0.05 vs Quartile 3 of HGI.

Additionally, we found that spot urine albumin/creatinine ratios (ACR), a known marker of kidney damage, was increased in the intermediate HGI group and, even more, in the high HGI group in comparison with the lowest quartile of HGI (Table 2). The difference in ACR levels between the lowest and the highest quartile of HGI remained statistically significant after adjusting for metabolic syndrome diagnosis, anti-hypertensive treatments and statin therapy in addition to gender and BMI (*P* ≤ 0.02).

**Figure 2:**
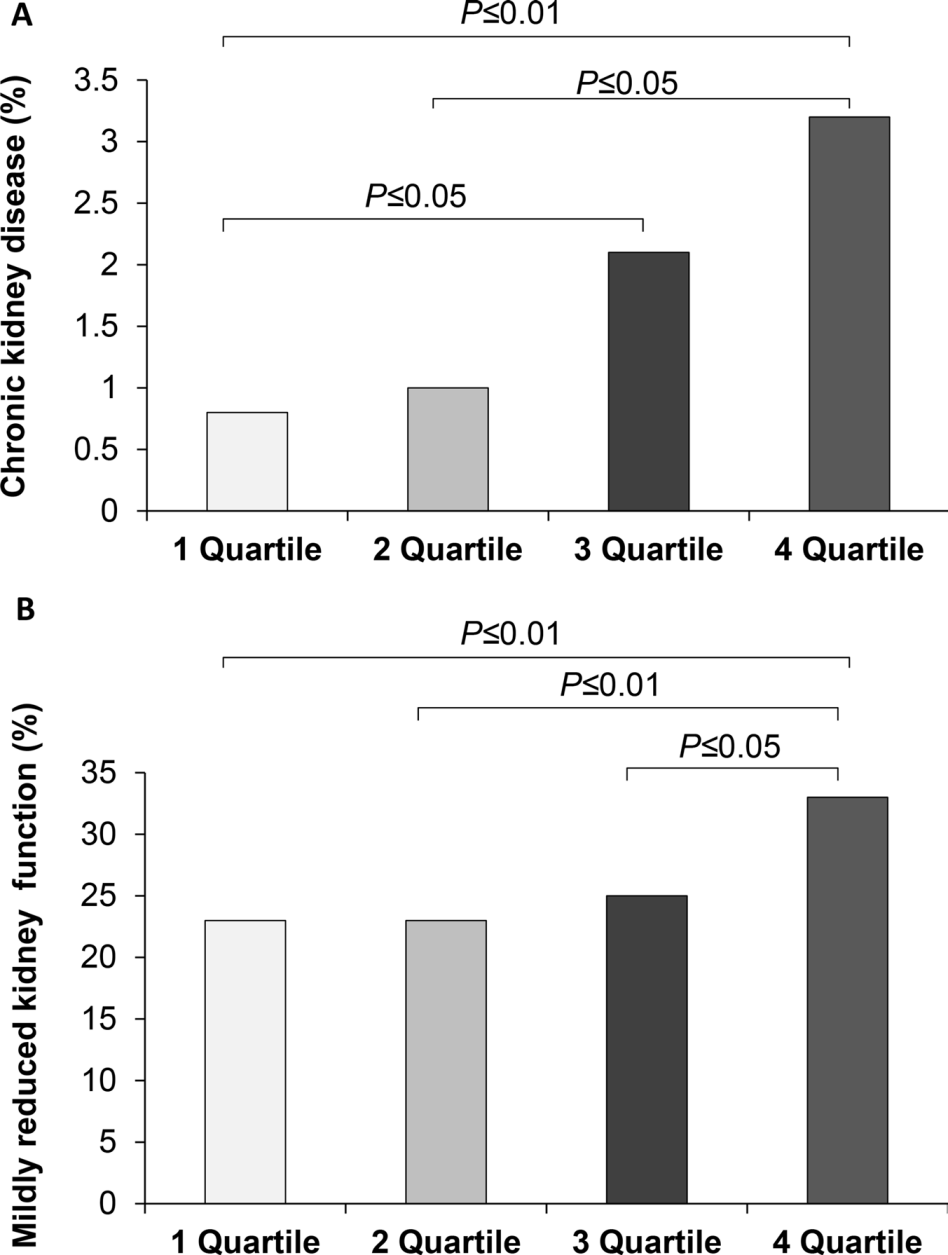
Prevalence of chronic kidney disease (**A**) and mildly reduced kidney function (**B**) in study population stratified in quartiles of hemoglobin glycation index (HGI).

Next, we evaluated the prevalence of chronic kidney disease (CKD, defined as eGFR<60 ml/min/1.73 m^2^) and mildly reduced kidney function (defined as eGFR 90–60 ml/min/1.73 m^2^) in the study population. As expected, since the whole study cohort was composed by nondiabetic subjects, the proportion of study participants with CKD was low (1.8%); however, we observed that the proportion of individuals with CKD was progressively increased in the intermediate and high HGI groups as compared to the lowest HGI group (*P* ≤ 0.05 and *P* ≤ 0.01 respectively) (Table 2 and Figure 2A). Of 1505 individuals examined, 391 had a mildly reduced kidney function (26%) and a significant increase in the proportion of subjects with mildly reduced kidney function was found in the highest HGI quartile in comparison to the other HGI groups (*P* ≤ 0.01 vs quartile 1 and 2 of HGI, *P* ≤ 0.05 vs quartile 3 of HIGI) (Table 2 and Figure 2B).

A logistic regression model adjusted for gender was used to estimate the association between HGI levels and the risk of impaired kidney function (Table 3). As compared to individuals in the lowest quartile of HGI, those in the highest quartile of HGI exhibited a significantly 3.6-fold increased OR of having CKD (95% CI: 1.13–11.24, *P* = 0.03). An increased OR of CKD was also observed in individuals in quartile 3 of HGI, even though it did not reach the threshold for statistical significance (2.06, 95% CI: 0.61–6.91, *P* = 0.24). Similar results were found when BMI, and smoking habit were included in the logistic regression model in addition to gender (Table 3). Subjects in the highest quartile of HGI had an increased risk of CKD in comparison to the lowest HGI group (OR: 3.44; 95% CI:1.01–12.54, *P =* 0.05) even after adjusting for metabolic syndrome diagnosis in addition to gender and smoking habit.

**Table 3:** Odds ratios (95% CI) by multiple logistic regression models for renal dysfunction in relation to HGI groups

		Chronic kidney disease eGFR < 60 ml/min/1.73 m^2^	
Study groups	OR	95% CI	*P*
**Model 1**			
Quartile 1 of HGI (reference category)	1	—	---
Quartile 2 of HGI	0.74	0.16–3.34	0.69
Quartile 3 of HGI	2.06	0.61–6.91	0.24
Quartile 4 of HGI	3.58	1.14–11.24	0.03
**Model 2**			
Quartile 1 of HGI (reference category)	1	—	---
Quartile 2 of HGI	0.69	0.15–3.13	0.63
Quartile 3 of HGI	2.00	0.59–6.83	0.27
Quartile 4 of HGI	3.47	1.07–11.21	0.04
Model 3			
Quartile 1 of HGI (reference category)	1	—	---
Quartile 2 of HGI	0.30	0.03–2.91	0.30
Quartile 3 of HGI	0.90	0.17–4.54	0.89
Quartile 4 of HGI	3.44	1.01–12.54	0.05
		**Mildly reduced kidney function** **eGFR 60–90 ml/min/1.73 m**^2^	
**Study groups**	**OR**	**95% CI**	***P***
**Model 1**			
Quartile 1 of HGI (reference category)	1	—	---
Quartile 2 of HGI	0.98	0.69–1.37	0.89
Quartile 3 of HGI	1.09	0.78–1.52	0.63
Quartile 4 of HGI	1.65	1.19–2.28	0.003
**Model 2**			
Quartile 1 of HGI (reference category)	1	—	---
Quartile 2 of HGI	0.95	0.66–1.38	0.81
Quartile 3 of HGI	1.07	0.74–1.56	0.71
Quartile 4 of HGI	1.52	1.05–2.18	0.03
**Model 3**			
Quartile 1 of HGI (reference category)	1	—	---
Quartile 2 of HGI	0.93	0.63–1.38	0.73
Quartile 3 of HGI	0.99	0.67–1.47	0.96
Quartile 4 of HGI	1.46	1.01–2.14	0.05

Model 1: adjusted for gender; Model 2: Model 1 + BMI, and smoking habit; Model 3: Model 1+ metabolic syndrome, and smoking habit.

Accordingly, individuals in the highest quartile of HGI had a significantly 1.6-fold increased OR of having a mildly reduced kidney function (95% CI: 1.19–2.28, *P* = 0.003) in comparison with the lowest HGI group in a logistic regression model adjusted for gender. Even after adjustment for BMI or metabolic syndrome diagnosis, and smoking habit in addition to gender the OR of subjects in the highest HGI quartile to have a mildly reduced kidney function remained significantly increased in comparison to individuals in the lowest quartile of HGI (Table 3).

## DISCUSSION

Given its strong relationship with plasma glucose levels, HbA1c is considered as the gold standard to assess glucose control and efficacy of therapy in patients affected by diabetes [9]. Moreover, assessment of HbA1c values is becoming commonly used also in subjects without history of diabetes as a diagnostic test for diabetes and prediabetes conditions [11–13]. Although it is widely recognized that plasma glucose concentrations are the major determinant of HbA1c levels, a discrepancy between HbA1c and other measures of glucose homeostasis including fasting plasma glucose [18], self-monitored blood glucose [14, 15, 19, 20], continuous glucose monitoring data [16, 21], and fructosamine [23, 24], has been reported and found to be consistent over time [22]. Remarkably, several studies have demonstrated that there are subjects with inappropriately low or high HbA1c levels relatively to their blood glucose concentrations not only among individuals affected by diabetes but also within nondiabetic population [14–22, 23, 24]. The existence of a biological inter-individual variation of HbA1c independent from circulating glucose levels suggests that factors other than glucose may influence hemoglobin glycation process, such as cellular permeability, 2,3-diphosphoglycerate concentration, levels and activity of glycolytic or deglycating enzymes [17, 22].

The HGI, which is calculated by subtracting from the observed HbA1c value the one expected on the basis of blood glucose concentrations, was developed in order to quantify the magnitude and the direction of the discordance commonly found between HbA1c and other measures of glucose control [14]. Higher HGI levels have been proposed to identify a phenotype of glucose metabolism characterized by an increased susceptibility to protein glycation, and tissue accumulation of advanced glycation end products (AGEs) [14, 25]. Notably, subjects affected by type 1 diabetes with higher HGI levels, calculated using self-glucose monitoring data, exhibited a higher prevalence and incidence of nephropathy and retinopathy [17]. Moreover, an analysis of patients with type 2 diabetes participating to the Action to Control Cardiovascular Risk in Diabetes (ACCORD) trial has shown a positive association between HGI, calculated using fasting glucose levels to predict HbA1c value, and the risk of both micro-vascular complications, and total mortality in the intensive treatment subgroup [18]. In addition, it has been recently reported that individuals with high HGI showed a significant increase in carotid intima media thickness, a well validated proxy of subclinical atherosclerosis [26]. Altogether these evidences highlight the crucial role of intracellular accumulation of glycated proteins, as estimated by high HGI, in the pathogenesis of organ damage caused by hyperglycemia.

Nevertheless, whether higher HGI levels may identify subjects with an increased risk of having an impaired kidney function among nondiabetic individuals is currently unsettled. Keeping in mind that an enhanced non-enzymatic glycation of intracellular proteins may alter their structure and function, and can promote AGEs accumulation resulting in tubular and glomerular injury [27, 28], we tested the hypothesis that a higher rate of non-enzymatic glycation of intracellular proteins, measured by HGI, is associated with kidney dysfunction also in nondiabetic subjects. In this cross-sectional study, we observed that nondiabetic subjects with higher HGI levels display a lower eGFR in comparison with individuals with lower HGI. Moreover, we found a positive association between HGI and albuminuria, a well-established marker of kidney damage and independent risk factor for cardiovascular morbidity and mortality [29,30]. Remarkably, we found that subjects with higher HGI levels displayed an increased risk of having CKD or a mildly reduced renal function in comparison to individuals with low HGI, even after adjustment for several confounders. In this regard, it should be noted that the risk of kidney dysfunction was found to be significantly increased only in subjects in the highest quartile of HGI, whereas it did not reach statistical significance in the third quartile of HGI. This observation supports the hypothesis that the link between HGI and impaired kidney function becomes significantly evident when the burden of intracellular glycated proteins exceeds the efficiency of compensatory factors, including glyoxalase system and NADPH-dependent enzymes, such as aldehyde reductase and aldose reductase, known to play an essential role in counteracting protein glycation and AGEs formation [31–33].

Importantly, levels of fasting, 1 hour and 2 hour post-load glucose were not significantly different between the study groups supporting the idea that the association between HGI and kidney dysfunction is not dependent of other measures of glucose metabolism in the extracellular compartment. Moreover, even though HbA1c levels may be affected by erythrocytes turnover, no significant difference in hemoglobin and hematocrit was observed between the study groups.

The underlying mechanism(s) by which high HGI may contribute to kidney dysfunction are still indefinite. Several cardio-metabolic risk factors, such as obesity, dyslipidemia, insulin resistance, chronic inflammation have been shown to represent important risk factors for the development of kidney disease [34–36]. Accordingly, we observed that high HGI is associated with a worse cardio-metabolic risk profile as demonstrated by increased values of BMI, waist circumference, total cholesterol and triglycerides, inflammatory biomarkers such as hsCRP and WBC count, and lower levels of HDL and insulin sensitivity in individuals with higher HGI levels as compared with those having low HGI.

Considering that HGI has been found to reflect the levels of AGEs in the tissues [25], a plausible candidate linking HGI with renal dysfunction may be represented by a raised burden of AGEs in the kidney of subjects with higher HGI levels. Non-enzymatic glycation of polypeptides, including hemoglobin, occurs via Maillard reaction, which is also involved in the formation of AGEs [25]. A large body of evidence has demonstrated that AGEs, directly or by binding specific receptors that recognize AGE-modified proteins (RAGE), may induce pro-inflammatory, oxidative and pro-fibrotic responses which are implicated in the development of kidney injury [27, 28, 37–39].

The present cross-sectional findings supporting the link between the individual tendency toward a raised intracellular protein glycation, estimated by higher values of HGI, and kidney dysfunction may have important clinical implications. Since kidney function impairment and higher concentrations of albumin in urine are independent predictors of cardiovascular morbidity and mortality in the general population [1–3, 30], an early identification of subjects with kidney damage is a crucial step to most effectively target intervention in an attempt to counteract the development of adverse clinical outcomes. It is well recognized that higher HbA1c levels are associated with kidney dysfunction not only in diabetic population but also in subjects without diabetes [6–8]. Our results suggest that HGI may be useful to identify among non-diabetic subjects with similar levels of HbA1c those with a “high glycation” phenotype having an increased risk of kidney disease.

The present study has several strengths and potential limitations that merit comment. The major strengths of the study include the large sample size, an extended clinical characterization with anthropometric and metabolic data collected by trained staff, the centralization of biochemical analyses including a rigorously standardized HbA1c measurement, the exclusion of conditions known to affect red cell survival, such as hemoglobinopathies and major blood loss, the use of oral glucose tolerance test (OGTT) data rather than fasting glucose alone as reported in previous studies [18].

Nevertheless, in interpreting our data some limitations should be taken in account. First all biochemical parameters, including plasma glucose during OGTT and HbA1c, were measured once. Even though this approach is commonly used in clinical research, between-individual variability of glucose homeostasis parameters may have led to some imprecision in the stratification of study population into HGI quartiles. Second, eGFR was used to identify and classify kidney disease. Isotopes clearance measurements may provide a more accurate evaluation of renal function; however, estimation of renal function by using MDRD equation is widely employed in large epidemiologic studies as well as in clinical practice, making our observations applicable to public health practice settings. Another limitation of the present study is the lack of a detailed diagnosis of kidney disease since no kidney biopsy or evaluation of specific kidney disease biomarkers was performed. Furthermore, insulin sensitivity was evaluated by the Matsuda index rather than by the gold standard technique euglycemic hyperinsulinemic clamp. However, it should be considered that euglycemic hyperinsulinemic clamp is a time-consuming and expensive procedure not feasible in large clinical studies, and Matsuda index has been shown to be highly correlated with whole-body insulin sensitivity assessed by euglycemic hyperinsulinemic clamp [40]. Additionally, we have recently demonstrated an association between HGI values with insulin sensitivity, assessed by the gold standard euglycemic hyperinsulinemic clamp [26]. Moreover, given the cross-sectional design of the study our observations suggest only an association between the rate of non-enzymatic glycation of intracellular proteins assessed by HGI and prevalent kidney dysfunction, and no causal relationship between the two phenomena may be established. Moreover all participants to the present study were White, and whether our results may be extendible to other ethnic groups, including Blacks, Hispanics, American Indians, that have been shown to exhibit higher levels of HbA1c than White subjects [41], warrants further investigations.

In conclusion HGI may represent a helpful tool to identify a subset of subjects harboring a greater risk of having kidney dysfunction not only among patients affected by diabetes mellitus, as reported by prior studies, but also within nondiabetic population.

## MATERIALS AND METHODS

We analyzed 1505 nondiabetic subjects participating to the CATAnzaro MEtabolic RIsk factors (CATAMERI) study, a cross-sectional study assessing cardio-metabolic risk factors in individuals carrying at least one risk factor including dysglycemia, overweight/obesity, hypertension, dyslipidemia, and family history for diabetes [26, 42, 43]. Exclusion criteria comprised: diagnosis of diabetes, history of any malignant disease, heart failure, gastrointestinal diseases associated with bleeding or malabsorption, autoimmune diseases, acute or chronic infections, acute or chronic pancreatitis, accumulation diseases such as amyloidosis and hemochromatosis, history of drug abuse, self-reporting alcohol consumption of > 20 g⁄day, positivity for antibodies to hepatitis C virus (HCV) or hepatitis B surface antigen (HBsAg), treatments able to modulate glucose metabolism, including corticosteroids and hypoglycemic agents. Anthropometrical parameters including BMI, waist circumference, blood pressure, body composition assessed by bioelectrical impedance, and biochemical data of study participants were collected after an over-night fasting. Each subject underwent a 75 g OGTT with 0, 30, 60, 90 and 120 min sampling for measurement of plasma glucose and insulin levels.

The protocol was approved by the Hospital ethical committee (Comitato Etico Azienda Ospedaliera “Mater Domini”) and all study participants gave written informed consent in accordance with principles of the Declaration of Helsinki.

### Analytical determinations

HbA1c levels were assessed with high performance liquid chromatography using a National Glycohemoglobin Standardization Program (NGSP) certified automated analyzer (Adams HA-8160 HbA1C analyzer, Menarini, Italy). Serum and urine creatinine concentrations were measured by an automated technique based on a Creatinine Jaffè compensated method for serum and urine (Roche Diagnostics) implemented in an auto-analyzer. Albuminuria was measured in fresh urine samples by immuneturbidimetry (Roche Diagnostics). An automated particle counter (Siemens Healthcare Diagnostics ADVIA^®^ 120/2120 Haematology System,Milan,Italy) was employed to measure hemoglobin, hematocrit, and WBC count.

Glucose, triglycerides, total and HDL cholesterol levels were assessed by enzymatic methods (Roche, Basel, Switzerland). Plasma insulin levels were measured with a chemiluminescence-based assay (Immulite^®^,Siemens Healthcare GmbH,Erlangen, Germany). Levels of hsCRP were assessed by an automated instrument (CardioPhase^®^ hsCRP,Milan,Italy).

### Calculation

Fasting plasma glucose and HbA1c data of the study population were used to estimate the linear relationship between the two parameters as described in previously published studies [18, 26]. The predicted level of HbA1c for each subject was calculated by inserting fasting plasma glucose concentration into the linear regression equation (HbA1c = 0.0158 * fasting glucose levels (mg/dl) +4.0311) [26]. HGI values were computed by subtracting the predicted levels of HbA1c from those observed, as previously described [18, 26]. Study participants were stratified into quartiles according to their HGI values.

The Matsuda index of insulin sensitivity was computed as follows: 10,000/square root of [fasting glucose (mmol/L) × fasting insulin (mU/L)] × [mean glucose × mean insulin during OGTT] [40].

eGFR was calculated by using the MDRD equation: eGFR = 175 × (Scr)^-1.154^ × (Age)^-0.203^ × (0.742 if female) where Scr is serum creatinine [44]. CKD was defined as eGFR < 60 ml/min/1.73 m^2^ and mildly reduced renal function was identified when eGFR was 90–60 ml/min/1.73 m^2^ [45].

Spot urine albumin (μg)/creatinine (mg) ratios (ACR) were calculated for 401 subjects, of whom albumin and creatinine levels in the morning urine samples were available [46].

Metabolic syndrome was defined as having three or more of the following criteria [47]: waist circumference > 102 cm in men and > 88 cm in women, triglycerides > 150 mg/dl or on treatment for elevated triglycerides, HDL < 40 mg/dl in men and < 50 mg/dl in women or on treatment for reduced HDL, blood pressure > 130/85 mmHg or on antihypertensive treatment, fasting glucose ≥ 100 mg/dl.

### Statistical analysis

Given their skewed distribution, triglycerides, HDL, hsCRP, fasting, 1-hour, and 2-hour post-load insulin were natural log transformed for statistical analyses. Continuous data are expressed as mean ± SD. χ^2^ test was employed to compare categorical variables. We used a general linear model to test pairwise differences in anthropometric and metabolic parameters among the study groups. A multivariate logistic regression analysis was performed to determine the association between the study groups and kidney function impairment. We considered statistically significant a two-sided *P* value ≤ 0.05. All analyses were carried out using SPSS software program Version 17.0 for Windows.

## Abbreviations

ACCORD: Action to Control Cardiovascular Risk in Diabetes
ACR: Spot urine albumin /creatinine ratios
AGEs: Advanced glycation end products
BMI: Body mass index
CATAMERI: CATAnzaro MEtabolic RIsk factors
CKD: Chronic kidney disease
eGFR: Estimated glomerular filtration rate
HbA1c: Glycated hemoglobin
HGI: Hemoglobin glycation index
HDL: High lipoprotein density
hsCRP: High sensitivity C reactive protein
OGTT: Oral glucose tolerance test
RAGE: Receptor of advanced glycation end products
WBC: White blood cell
